# Persistent Cryptococcal Brain Infection despite Prolonged Immunorecovery in an HIV-Positive Patient

**DOI:** 10.1155/2014/164826

**Published:** 2014-03-05

**Authors:** Tom Wingfield, Jo Baxter, Amit Herwadkar, Daniel du Plessis, Tom J. Blanchard, F. Javier Vilar, Anoop Varma

**Affiliations:** ^1^Section of Infectious Diseases & Immunity and Wellcome Trust, Imperial College Centre for Global Health Research, Imperial College London Hammersmith Hospital Campus, 150 Du Cane Road, London W12 0NN, UK; ^2^The Monsall Infection Unit, Regional Department of Infectious Diseases and Tropical Medicine, North Manchester General Hospital, Delaunays Road, Manchester M8 5RB, UK; ^3^Department of Neuroradiology, North Manchester General Hospital, Delaunays Road, Manchester M8 5RB, UK; ^4^Department of Neuropathology, Salford Royal Hospital, Stott Lane, Salford M6 8HD, UK; ^5^Department of Neurology, North Manchester General Hospital, Delaunays Road, Manchester M8 5RB, UK; ^6^Department of Neurology, Salford Royal Hospital, Stott Lane, Salford M6 8HD, UK

## Abstract

*Background.* HIV-positive people starting combined antiretroviral therapy may develop immune reconstitution to latent or treated opportunistic infections. Immune reconstitution to cerebral Cryptococcus is poorly understood and can be fatal. *Case Presentation.* A 33-year-old Zimbabwean female presented with cryptococcal meningitis and newly diagnosed HIV with a CD4 count of 51 cells/**μ**L (4%). She was treated with amphotericin and flucytosine. Combined antiretroviral therapy was started four weeks later and she showed early improvement. However, over the ensuing 18 months, her clinical course was marked by periodic worsening with symptoms resembling cryptococcal meningitis despite having achieved CD4 counts ≥400 cells/**μ**L. Although initially treated for relapsing cryptococcal immune reconstitution syndrome, a brain biopsy taken 17 months after initial presentation showed budding Cryptococci. *Conclusion.* This unusually protracted case highlights the difficulties in differentiating relapsing cryptococcal meningitis from immune reconstitution and raises questions concerning the optimum timing of initiation of combined antiretroviral therapy in such patients.

## 1. Introduction

We describe a patient with cryptococcal meningitis and an atypical immune reconstitution syndrome (IRIS) with multiple relapses over 18 months. We explore the diagnostic and therapeutic challenges surrounding the recognition and management of such relapsing cryptococcal disease. We also elaborate on how this rare case provided further insight into the pathological progression of IRIS, a phenomenon still not fully understood.

## 2. Case

In July 2005, a 33 year old Zimbabwean female, resident in the UK for 8 years, was admitted with a week history of fever, headache, and neck stiffness. She had no past medical history of note. Systemic examination showed fever and meningism but no focal neurological deficits. Blood tests revealed mild anaemia, lymphopenia, and raised C-reactive protein. An HIV test was positive with CD4 count of 51 cells/*μ*L (4%). CT head without contrast showed marked meningeal inflammation but no focal lesions. A lumbar puncture (LP) revealed raised opening pressure (OP) of 27 cms H_2_0, lymphocyte count (30/cu mm), protein (1.33 g/L), and CSF: serum glucose ratio of 50%. Cerebral spinal fluid (CSF) microscopy showed multiple yeast with high cryptococcal antigen (CRAG) titre of >1 : 25,600. CSF Acid-alcohol fast bacilli (AAFB), toxoplasmosis, and viral screens were negative. The patient was started on standard amphotericin with flucytosine and prophylactic cotrimoxazole.

**Figure 1:**
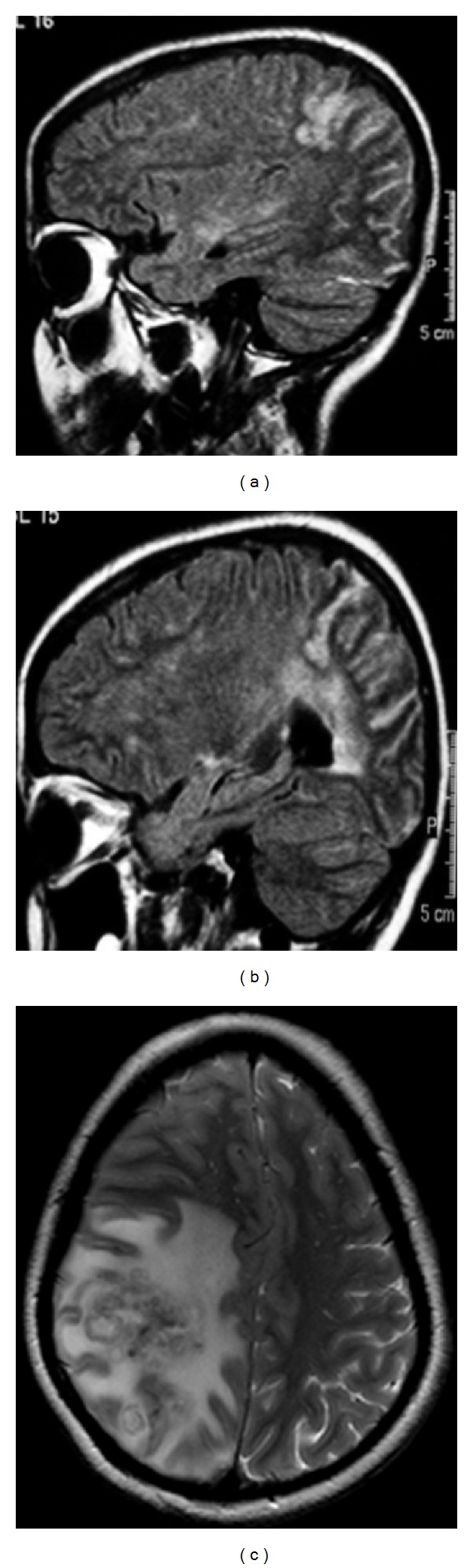
(a) MRI brain 5 days into admission in August 2005 showing right-sided occipitoparietal leptomeningeal inflammation. (b) MRI brain on readmission December 2005, 4 months after initial presentation showing worsening right-sided occipitoparietal leptomeningeal inflammation. (c) T2-weighted MRI brain in December 2006, 16 months after initial presentation showing large clusters of ring-enhancing lesions and leptomeningeal inflammation in the right occipitoparietal area with oedema and midline shift. Also seen but not shown on this cross-section were smaller clustered ring-enhancing lesions in the right thalamus and left frontal and temporal lobes.

On day five of therapy, she began to complain of increasing headache and neck stiffness. A right-sided sixth nerve palsy was noted as a new neurological sign. An MRI brain showed changes in keeping with chronic meningitis and occipitoparietal changes (Figure 1(a)). Repeat LP revealed OP of 40 cms H_2_0 with persistently high protein and lymphocyte count. Over the next two weeks, the patient required a lumbar drain to alleviate excruciating pressure-related headaches.

CSF cultures revealed *Cryptococcus neoformans *sensitive to amphotericin B (Minimum Inhibitory Concentration “MIC” 0.125 mg/L) and itraconazole (MIC 0.03 mg/L) and intermediate to fluconazole (MIC 4.0 mg/L) and flucytosine (MIC 8.0 mgL). Amphotericin and flucytosine were continued for four weeks and then switched to oral fluconazole 800 mg once daily. This prolonged initial phase was based on symptom severity, high fungal burden, and relatively high MIC to fluconazole. A repeat MRI head showed regression of the previous changes.

Four weeks into cryptococcal treatment, combined antiretroviral therapy (cART) was initiated with Kivexa (abacavir and lamivudine) and efavirenz. The patient then developed a new, persistent headache and subsequent LPs showed raised OPs, lymphocytes, and protein. Yeast cells persisted in CSF for 3 months after starting amphotericin and flucytosine and CSF cultures remained positive until September 2005 (2 months after treatment start).

By October 2005, CSF cultures were negative for Cryptococcus and the patient had improved clinically. VL was undetectable and CD4 counts had risen minimally from 51 (4%) to 67 cells/*μ*L (6%). She was discharged home on fluconazole (800 mg OD), cotrimoxazole, and cART.

The patient then went on to have a difficult clinical course with recurrent admissions over the subsequent 14 months (Table 1).

## 3. Discussion

Cryptococcosis continues to be a major opportunistic pathogen in HIV-positive patients despite global upscaling of cART [1]. HIV causes depletion of T cell immunity allowing infection by, most commonly, *Cryptococcus neoformans *(*var grubii* or *neoformans*) [2, 3]. Cerebral cryptococcal infection remains the commonest cause of meningitis in areas of sub-Saharan Africa [4].

Current guidelines for therapy for cryptococcal meningitis suggest amphotericin B at 0.7–1 mg/kg/day (or liposomal amphotericin B if renally impaired) combined with flucytosine 100 mg/kg/day switched to oral fluconazole after at least two weeks or once CSF sterility has been achieved. Fluconazole is then continued for a further 6 to 12 months or until CD4 count is above 250 cells/*μ*L for 6 months [1, 2, 5–10]. Predictive markers of mycological failure have been found to be disseminated cryptococcal disease, high CSF CRAG titres and initial treatment lacking flucytosine [2, 11].

Through immune restoration, cART has decreased morbidity and mortality from AIDS-associated opportunistic infections (OIs) [12, 13]. Although still not fully understood, IRIS represents a dysregulated immune response to pathogen-specific antigens occurring especially in HIV positive patients with advanced immunodeficiency commencing cART [14–16]. IRIS incidence in such patients varies from 10 to 32% [17–19]. IRIS can be subdivided into either “paradoxical” reactions which are a response to pathogen-specific antigens despite the pathogen itself being nonviable, or “unmasking” reactions which are a response to infections that were subclinical prior to cART [14, 15, 19]. Both types of IRIS are most common in the first 3 months after initiating cART but paradoxical IRIS may present much later, in some cases up to 2 years after initiation [10, 14]. Multiple manifestations of IRIS have been reported, including mycobacterium avium intracellulare lymphadenitis, pulmonary and neurological tuberculosis, and cryptococcal meningitis [14, 15]. Risk factors for IRIS include disseminated OI disease; recent OI treatment; low baseline CD4 with rapid rise after starting cART; and high baseline HIV VL with rapid decline after starting cART [14, 17, 20, 21].

Paradoxical IRIS in HIV-positive patients with previously treated cryptococcal disease has been estimated between 4 and 30% and is associated with an exaggerated T-cell mediated production of interferon-gamma to pathogen specific antigens [10, 12, 18, 22, 23]. The most common presentations of cryptococcal IRIS are either meningitis or lymphadenitis [24]. This marked inflammatory response manifests itself clinically, with fever, lymphadenopathy, and meningism due to raised ICP; microbiologically, with high protein levels and CSF white cell counts including polymorphonuclear cells; neuroradiologically, with extensive abnormal contrast enhancement; and histologically, with granulomas composed mainly of macrophages (containing inert cryptococci) and high levels of CD8+ cytotoxic lymphocytes [10, 25–28].

Our patient presented with cryptococcal meningitis as an AIDS-defining illness. She had a low CD4 count of 51 cells/*μ*L (4%) and a high CSF CRAG titre of >1 : 25,600, visible yeast on microscopy, and subsequent positive fungal cultures. Her CSF remained culture positive for Cryptococcus until eight weeks after starting high-dose fluconazole, a total of 12 weeks after presentation, indicating a massive cryptococcal burden. Despite oral fluconazole, she had florid recrudescence of her symptoms at 1-2 months into cART with focal neurology, worsening MRI changes, biopsy-proven live Cryptococcus, and a good response to steroid therapy, typical of an unmasking IRIS [10, 25, 29]. This initial presentation was in keeping with the literature which highlights high baseline fungal burden (high blood or CSF CRAG), high fungal burden at end of amphotericin B induction treatment, low initial CD4 count, and early initiation of ART (less than 1-2 months from diagnosis of cryptococcal meningitis) as risk factors for cryptococcal IRIS [30–32]. Our patient, however, had by this time achieved only a minimal rise in her CD4 count (51 to 67 cells/*μ*L) and, therefore, hypothetically minimal immune reconstitution.

**Figure 2:**
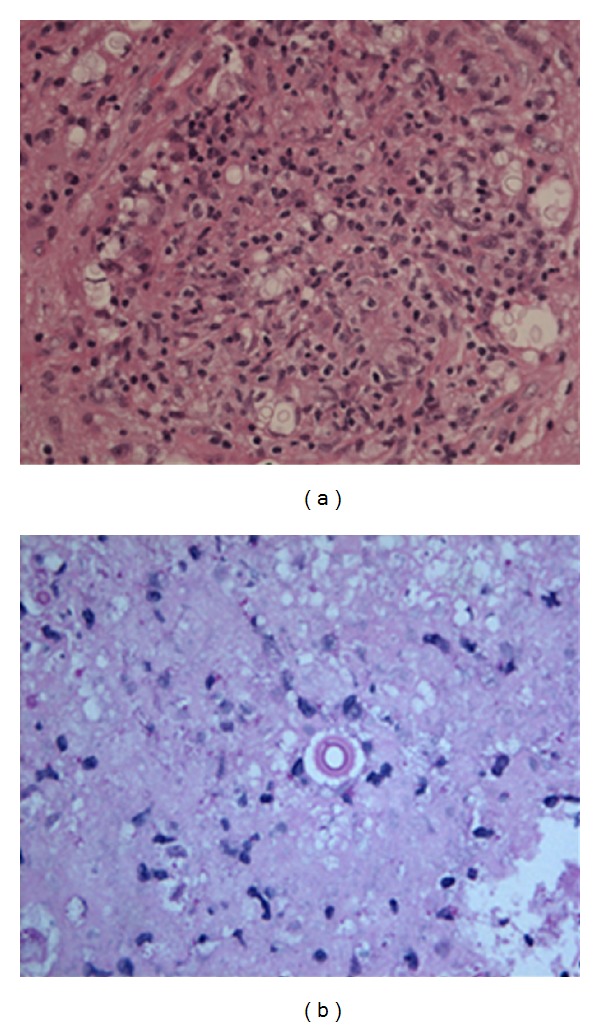
(a) Brain biopsy taken in December 2005, 4 months after initial presentation showing reactive gliosis and focally distended perivascular spaces containing Cryptococcus. (b) Brain biopsy taken in December 2006, 16 months after initial presentation showing encapsulated, budding yeast forms, scattered singly with some surrounding necrosis and moderate chronic inflammation. No granulomata were seen.

The AIDS Clinical Trials Group study 1564 reported benefits for starting cART early in the context of acute OIs (12% Cryptococcus) [33]. It is, however, our patient's later presentations that provide contrast to existing guidelines for the management of cryptococcal IRIS. Eighteen months after her initial presentation, 11 months after achieving CSF sterility, and 2 months after finishing a year's course of fluconazole and tapered steroids, she represented with a new acute syndrome with significant worsening in MRI appearance and visible budding yeast on brain biopsy (see Figure 2), indicative of active infection. By this point, her CD4 count had risen substantially to 437 cells/*μ*L (18%) and this, in conjunction with the profound inflammatory reaction and live budding Cryptococcus, may have indicated a relapse of cryptococcal IRIS or disease. It appears that our patient was unable to arrest fungal replication despite what appeared to be a good peripheral blood CD4 count and supposed “immune reconstitution”. This raises questions concerning the quality of the CD4 response, compartmentalisation of CD4 immunity, and the correlation of a rise in peripheral CD4 count with activity of CNS immunity [34–36].

This unusual case illustrates that HIV-associated cryptococcal IRIS, especially in severely immunocompromised patients with high burden of organism, can be difficult to distinguish from recurrent cryptococcal meningitis and that the role of MIC in clinical practice for cryptococcal meningitis remains to be defined [37]. Regarding MIC, our experience with this patient does not correlate with the recently published guidelines [38] for the management of cryptococcal meningitis: in particular continuing fluconazole treatment and repeated brain biopsies were essential for management despite the CD4 count increasing above 100 cells/*μ*L. Corticosteroids for IRIS caused substantial morbidity, but there is no established alternative and possible therapy with thalidomide would not have been safe in this patient of childbearing potential. Were our patient to have continued to relapse with active cryptococcal disease, we would have needed to consider suppressive therapy with newer broad spectrum azoles such as voriconazole or posaconazole (echinocandins have no activity against Cryptococcus) [38].

In order to prevent relapse of symptoms, patients such as ours may require prolongation of amphotericin B and flucytosine induction therapy and subsequent fluconazole and steroid maintenance therapy, beyond that recommended by existing guidelines [39]. This case highlights the difficulty in differentiating cryptococcal meningitis from cryptococcal IRIS [40, 41] and emphasizes the need for further evidence on the optimal time to start ART in HIV-positive patients presenting with cryptococcal meningitis as highlighted by a recent Cochrane review [41].

## Figures and Tables

**Table 1:** The patient's relapsing clinical course with setting, symptoms, signs, investigations, imaging, and treatment choices.

Months after 1st presentation	Presenting complaint and potential precipitant	LP results	HIV parameters	MRI results	Brain biopsy	Treatment and length of stay
3 Inpatient	Fever and meningism No obvious precipitant	↑OP/lymphocytes/protein CRAG 1 : 100 and yeast cells seen Culture negative	CD4 76 cells/*μ*L (6%) HIV VL 601 copies/mL	—	—	Fluconazole changed to itraconazole due to MIC Steroids started and patient discharged after two week stay on slowly tapering prednisolone dose

4 Inpatient	Two focal seizures with secondary generalization. New neurological signs: RUL spastic catch, BL lower limb spasticity and ↑right plantar Patient ran out of prednisolone	↑OP/lymphocytes/protein CRAG 1 : 32 Yeast cells not seen Culture negative	CD4 67 cells/*μ*L (5%) HIV VL 95 copies/mL	Worsening right occipito-parietal focal meningeal inflammation (Figure 1(b))	Reactive gliosis and focally distended perivascular spaces containing cryptococcus (Figure 2(a)) No other organisms were identified or cultured	Itraconazole dose increased and prednisolone reinstated Lamotrigine commenced Discharged after two week in-patient stay on itraconazole, prednisolone, and lamotrigine

8 Outpatient	Asymptomatic	—	CD4 190 cells/*μ*L (9%) HIV VL 40 copies/mL	—	—	Itraconazole changed to fluconazole Prednisolone tapered by 1 mg per week

15 Outpatient	Non-attendance at clinic Self-cessation of fluconazole despite good compliance otherwise	—	CD4 473 cells/*μ*L (18%) HIV VL < 40 copies/mL	—	—	Fluconazole not restarted at this point

16 Inpatient	Left-sided focal motor seizures with secondary generalization Neurological examination normal, no headache 10/40 pregnant but miscarried	—	—	—	—	Lamotrigine increased and clobazam added to anti-epileptic regimen Efavirenz substituted with lopinavir plus ritonavir for ↑CNS penetration Total inpatient stay of five days

17 Inpatient	Headache, nausea and increasing unsteadiness Clinical left sided dysdiadochokinesis with unsteady gait and inability to walk heel-to-toe.	First: ↑lymphocyte count (88/cu mm)/↑protein CRAG/viral screen/AAFB No observable yeast Culture negative Second: Improving lymphocyte count (14/cu mm), all else as per first LP	CD4 560 cells/*μ*L (24%) HIV VL < 40 copies/mL	Large clusters of ring-enhancing lesions and leptomeningeal inflammation in R occipito-parietal area with oedema and midline shift. Smaller clustered ring-enhancing lesions in R thalamus and L frontal and temporal lobes (Figure 1(c))	Brain biopsy showed cryptococcal, encapsulated, budding yeast forms, scattered singly with some surrounding necrosis and moderate chronic inflammation No granulomata were seen (Figure 2(b)) No other organisms were identified or cultured	Ambisome 3 mg/kg once daily, flucytosine and high dose dexamethasone commenced Ambisome/flucytosine stopped when CSF CRAG result negative Ambisome restarted without flucytosine when brain biopsy results became available One month stay, discharged on 600 mg fluconazole orally (changed to 400 mg in OPD clinic 6 weeks later) and a reducing steroid dose

40 Outpatient	Full resolution of neurological symptoms No further seizures	—	—	Resolution of MRI features	—	Self-discontinued fluconazole. Since discharge developed steroid-induced DM and avascular necrosis

Abbreviations: VL: viral load; L: left; R: right; BL: bilateral; ↑: raised above normal values; CNS: entral nervous system; DM: diabetes mellitus; CRAG: cryptococcal antigen; —: not performed.

## References

[1] Jarvis JN, Dromer F, Harrison TS, Lortholary O (2008). Managing cryptococcosis in the immunocompromised host. *Current Opinion in Infectious Diseases*.

[2] Dromer F, Mathoulin-Pélissier S, Launay O, Lortholary O (2007). Determinants of disease presentation and outcome during cryptococcosis: the crypto A/D study. *PLoS Medicine*.

[3] Jarvis JN, Harrison TS (2007). HIV-associated cryptococcal meningitis. *Journal of Acquired Immune Deficiency Syndromes*.

[4] Scarborough M, Gordon SB, Whitty C (2007). Corticosteroids for bacterial meningitis in adults in sub-Saharan Africa. *The New England Journal of Medicine*.

[5] Pattman R, Snow M, Handy P, Sankar KN, Elawad B (2005). Cryptococcal meningitis. *Oxford Handbook of Genitourinary Medicine, HIV, and AIDS*.

[6] Mandal B, Wilkins E, Dunbar E, Mayon-White R (2004). Cryptococcal meningitis. *Lecture Notes on Infectious Diseases*.

[7] Bicanic T, Wood R, Meintjes G (2008). High-dose amphotericin B with flucytosine for the treatment of cryptococcal meningitis in HIV-infected patients: a randomized trial. *Clinical Infectious Diseases*.

[8] van der Horst C, Saag M, Cloud G (1997). Treatment of cryptococcal meningitis associated with acquired immunodeficiency syndrome. *The New England Journal of Medicine*.

[9] Segal B, Kwon-Chung J, Walsh T (2006). Immunotherapy for fungal infections. *Clinical Infectious Diseases*.

[10] Bicanic T, Wood R, Meintjes G (2008). High-dose amphotericin B with flucytosine for the treatment of cryptococcal meningitis in HIV-infected patients: a randomized trial. *Clinical Infectious Diseases*.

[11] Dromer F, Bernede-Bauduin C, Guillemot D, Lortholary O (2008). Major role for amphotericin B-flucytosine combination in severe cryptococcosis. *PLoS ONE*.

[12] Lawn SD, Harries AD, Anglaret X, Myer L, Wood R (2008). Early mortality among adults accessing antiretroviral treatment programmes in sub-Saharan Africa. *Journal of Acquired Immune Deficiency Syndromes*.

[13] Palella F, Delaney K, Moorman A (1998). Declining morbidity and mortality among patients with advanced human immunodeficiency virus infection. *The New England Journal of Medicine*.

[14] Shelburne SA, Hamill RJ, Rodriguez-Barradas MC (2002). Immune reconstitution inflammatory syndrome: emergence of a unique syndrome during highly active antiretroviral therapy. *Medicine*.

[15] Mayer KH, French M (2009). Immune reconstition inflammatory syndrome: a reappraisal. *Clinical Infectious Diseases*.

[16] French M, Price P, Stone S (2004). Immune restoration disease after antiretroviral therapy. *Journal of Acquired Immune Deficiency Syndromes*.

[17] Murdoch D, Venter W, Feldman C, van Rie A (2008). Incidence and risk factors for the immune reconstitution inflammatory syndrome in HIV patients in South Africa: a prospective study. *Journal of Acquired Immune Deficiency Syndromes*.

[18] Shelburne S, Visnegarwala F, Darcourt J (2005). Incidence and risk factors for immune reconstitution inflammatory syndrome during highly active antiretroviral therapy. *Journal of Acquired Immune Deficiency Syndromes*.

[19] French M, Lenzo N, John M (2000). Immune restoration disease after the treatment of immunodeficient HIV-infected patients with highly active antiretroviral therapy. *HIV Medicine*.

[20] Breton G, Duval X, Estellat C (2004). Determinants of immune reconstitution inflammatory syndrome in HIV type 1-infected patients with tuberculosis after initiation of antiretroviral therapy. *Clinical Infectious Diseases*.

[21] Manabe Y, Campbell J, Sydnor E, Moore RD (2007). Immune reconstitution inflammatory syndrome: risk factors and treatment implications. *Journal of Acquired Immune Deficiency Syndromes*.

[22] Bourgarit A, Carcelain G, Martinez V (2006). Explosion of tuberculin-specific Th1-responses induces immune restoration syndrome in tuberculosis and HIV co-infected patients. *Journal of Acquired Immune Deficiency Syndromes*.

[23] Tan D, Yong Y, Tan H (2008). Immunological profiles of immune restoration disease presenting as mycobacterial lymphadenitis and cryptococcal meningitis. *HIV Medicine*.

[24] Skiest DJ, Hester LJ, Hardy RD (2005). Cryptococcal immune reconstitution inflammatory syndrome: report of four cases in three patients and review of the literature. *Journal of Infection*.

[25] Jenny-Avital E, Abadi M (2002). Immune reconstitution cryptococcosis after initiation of successful highly active antiretroviral therapy. *Clinical Infectious Diseases*.

[26] Cinti S, Armstrong W, Kauffman C (2001). Case report. Recurrence of increased intracranial pressure with antiretroviral therapy in an AIDS patient with cryptococcal meningitis. *Mycoses*.

[27] King M, Perlino C, Cinnamon J, Jernigan JA (2002). Paradoxical recurrent meningitis following therapy of cryptococcal meningitis: an immune reconstitution syndrome after initiation of highly active antiretroviral therapy. *International Journal of STD and AIDS*.

[28] Gray F, Bazille C, Adle-Biassette H, Mikol J, Moulignier A, Scaravilli F (2005). Central nervous system immune reconstitution disease in acquired immunodeficiency syndrome patients receiving highly active antiretroviral treatment. *Journal of NeuroVirology*.

[29] Woods M, MacGinley R, Eisen DP, Allworth AM (1998). HIV combination therapy: partial immune restitution unmasking latent cryptococcal infection. *Journal of Acquired Immune Deficiency Syndromes*.

[30] Bicanic T, Meintjes G, Rebe K (2009). Immune reconstitution inflammatory syndrome in HIV-associated cryptococcal meningitis: a prospective study. *Journal of Acquired Immune Deficiency Syndromes*.

[31] Lortholary O, Fontanet A, Mémain N, Martin A, Sitbon K, Dromer F (2005). Incidence and risk factors of immune reconstitution inflammatory syndrome complicating HIV-associated cryptococcosis in France. *Journal of Acquired Immune Deficiency Syndromes*.

[32] Sungkanuparph S, Jongwutiwes U, Kiertiburanakul S (2007). Timing of cryptococcal immune reconstitution inflammatory syndrome after antiretroviral therapy in patients with AIDS and cryptococcal meningitis. *Journal of Acquired Immune Deficiency Syndromes*.

[33] Zolopa AR, Andersen J, Komarow L (2009). Early antiretroviral therapy reduces AIDS progression/death in individuals with acute opportunistic infections: a multicenter randomized strategy trial. *PLoS ONE*.

[34] Mavigenr M, Delobel P, Cazabat M (2009). HIV-1 residual viremia correlates with persistent T-cell activation in poor immunological responders to combination antiretroviral therapy. *PLoS ONE*.

[35] Pierson T, McArthur J, Siliciano R (2000). Reservoirs for HIV-1: mechanisms for viral persistence in the presence of antiviral immune responses and antiretroviral therapy. *Annual Review of Immunology*.

[36] Haggerty C, Pitt E, Siliciano R (2006). The latent reservoir for HIV-1 in resting CD4+ T cells and other viral reservoirs during chronic infection: insights from treatment and treatment-interruption trials. *Current Opinion in HIV & AIDS*.

[37] Manosuthi W, Sungkanuparph S, Thongyen S (2006). Antifungal susceptibilities of cryptococcus neoformans cerebrospinal fluid isolates and clinical outcomes of cryptococcal meningitis in HIV-infected patients with/without fluconazole prophylaxis. *Journal of the Medical Association of Thailand*.

[38] Perfect JR, Dismukes WE, Dromer F (2010). Clinical practice guidelines for the management of cryptococcal disease: 2010 update by the Infectious Diseases Society of America. *Clinical Infectious Diseases*.

[39] Lodha A, Haran M (2009). Is it recurrent cryptococcal meningitis or immune reconstitution inflammatory syndrome?. *International Journal of STD & AIDS*.

[40] Jhamb R, Kashyap B, Das S, Berry N, Garg A (2013). Symptomatic relapse of HIV-associated cryptococcal meningitis: recurrent cryptococcal meningitis or cryptococcus-related immune reconstitution inflammatory syndrome?. *International Journal of STD & AIDS*.

[41] Njei B, Kongnyuy EJ, Kumar S, Okwen MP, Sankar MJ, Mbuagbaw L (2013). Optimal timing for antiretroviral therapy initiation in patients with HIV infection and concurrent cryptococcal meningitis. *The Cochrane Database of Systematic Reviews*.

